# Intestinal obstruction after surgery for congenital biliary dilatation in children: diagnosis and management

**DOI:** 10.3389/fped.2025.1558884

**Published:** 2025-03-25

**Authors:** Zhen-sheng Liu, Jian Bian, Yong Yang, De-cheng Wei, Shi-qin Qi

**Affiliations:** Department of General Surgery, Anhui Provincial Children's Hospital, Hefei, Anhui Province, China

**Keywords:** congenital biliary dilatation, intestinal obstruction, children, choledochal cyst, internal hernia

## Abstract

**Objective:**

To analyze etiologies and management of postoperative intestinal obstruction following surgery (exeision of the dilated bile duet and Roux-enY hepaticojejunostomy) for congenital biliary dilatation (CBD) in children.

**Methods:**

A single-institution retrospective review was conducted on 475 patients who underwent Roux-en-Y hepaticojejunostomy following complete excision of the dilated bile duct. Among the cohort, nine patients underwent reoperation for intestinal obstruction. The perioperative data of these cases were thoroughly analyzed.

**Results:**

The cohort (8F:1M) developed obstruction 20 days-8.8 years postoperatively. Primary etiologies included internal hernias (Petersen's:2, transverse mesocolic:3, Brolin's:1), biliary-jejunal loop torsion (1), and adhesions (2). Three patients underwent redo biliary-enteric anastomosis secondary to Roux-en-Y loop necrosis. Cross-sectional imaging in children with internal hernia or Roux-en-Y volvulus demonstrated distended, fluid-filled biliary-jejunal loops at the porta hepatis. Surgical indications for intestinal obstruction included peritoneal signs, aggravated abdominal pain, and failure of conservative treatment. Two children with intestinal obstruction had abnormal liver function tests preoperatively.

**Conclusion:**

Internal hernias (particularly within the internal hernia triangle) are the predominant cause of post-CBD surgery obstruction. Cross-sectional imaging shows high diagnostic sensitivity. Given the higher likelihood of internal hernia as a cause of post-CBD surgery obstruction and its rapid progression to Roux limb necrosis, early surgical intervention should be considered.

## 1 Introduction

**Figure 1:**
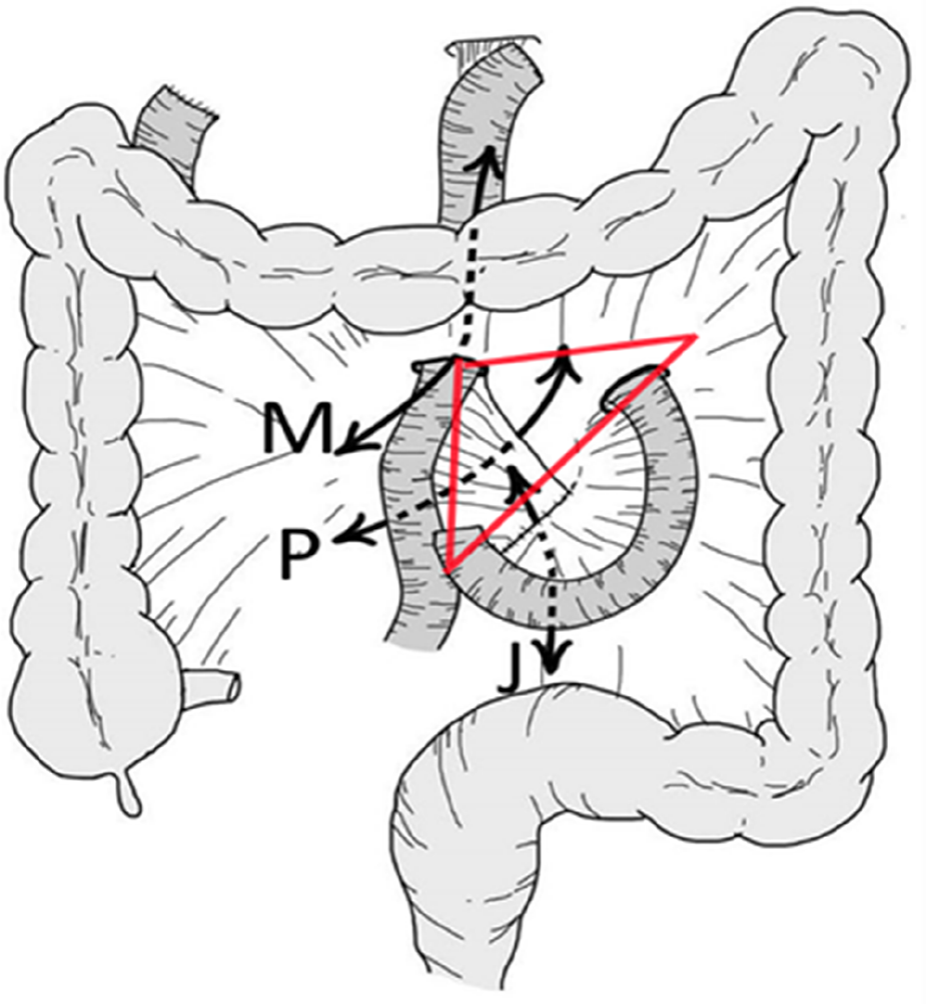
P: defect between Roux limb mesentery and transverse mesocolon (Petersen space), J: defect at jejunojejunostomy (Brolin space), and M: defect in transverse mesocolon (retrocolic approach) (3, 20, 21). The red triangle represents the internal hernia triangle.

Congenital Biliary Dilatation (CBD) is a congenital condition characterized by abnormal dilation of the biliary system. The etiology remains unclear, although it is widely believed to be associated with abnormal pancreaticobiliary junction. Common symptoms include recurrent abdominal pain, jaundice, vomiting, and pancreatitis. Severe complications, such as biliary perforation or cholangiocarcinoma, may lead to life-threatening consequences (1). The standard surgical treatment for congenital biliary dilatation (CBD) requires resection of the dilated biliary tract followed by retrocolic Roux-en-Y hepaticojejunostomy reconstruction (2). This procedure creates three mesenteric defects, as shown in Figure 1. The Petersen space is the region between the biliary-jejunal loop and the transverse mesocolon; the Brolin space refers to the area between the mesenteric attachments of the two limbs of the Y-shaped jejunal anastomosis; the transverse mesocolic defect is the opening through which the biliary-jejuna loop passes through the mesocolon (3). Intestinal obstruction is a known complication following CBD surgery. If improperly managed, it can lead to severe consequences. However, there is limited research on children who suffer from intestinal obstruction after CBD surgery and necessitate surgical intervention. This study retrospectively analyzes the clinical data of 9 pediatric patients who underwent operation for intestinal obstruction after CBD surgery, aiming to summarize the diagnostic and therapeutic experiences.

## 2 Methods

### 2.1 Inclusion criteria

We conducted a retrospective analysis of clinical data from 475 pediatric patients with CBD treated at Anhui Provincial Children's Hospital between July 1, 2013, and July 1, 2024. Among these, 9 patients underwent operation due to intestinal obstruction. The clinical data from both surgeries were collected in 9 cases. Inclusion criteria: (1). Age <18 years. (2). Surgery for CBD performed at Anhui Provincial Children's Hospital. (3). Surgery for intestinal obstruction performed at our hospital or affiliated medical institutions, and the clinical data is accessible. Exclusion criteria: (1). Previous history of abdominal surgery unrelated to CBD. (2). Incomplete clinical data. In one case, intestinal obstruction surgery was performed at an affiliated institution, and we obtained relevant clinical and imaging data by contacting the primary physician.

### 2.2 Observational parameters

Perioperative data were collected from 9 pediatric patients who underwent surgery for intestinal obstruction following CBD surgery. The data collected include demographic information (gender and age), details of the surgery for CBD (surgical method and perioperative complications), and clinical findings related to intestinal obstruction (symptoms, preoperative liver function tests, upright abdominal x-ray, and enhanced CT or MRI). For intestinal obstruction surgery, the indications, surgical techniques, causes of obstruction, duration of conservative treatment, and the interval between the two surgeries were also documented.

### 2.3 Follow-up

Postoperative follow-up was conducted through outpatient visits at 1, 3, 6, and 12 months, followed by visits every six months thereafter. Each follow-up included assessments of liver function, complete blood count, and abdominal ultrasonography focusing on the biliary tree and any potential complications related to the surgery.

## 3 Results

### 3.1 Initial surgery details

The initial surgical data for the 9 patients are shown in Table 1. Among these, 3 cases underwent open surgery for CBD, while 6 cases were performed laparoscopically. Postoperatively, 3 patients developed bile leakage, which was treated with laparoscopic drainage tube placement. All 9 patients had hepaticojejunostomy via a retrocolic route. During surgery, the Petersen space was left open, the Brolin space closed with continuous absorbable sutures, and the mesocolic defects closed with interrupted absorbable sutures in 5 patients. Mesocolic defects were left unclosed in 4 patients, 3 of whom developed colonic mesenteric hernias.

**Table 1:** Clinical data of the initial surgery.

Case No.	Excision of the dilated bile duct and hepaticojejunostomy Roux-en-Y anastomosis	Petersen space	Brolin space	Transverse mesocolic defect	Perioperative complication
1	open	Not closed	C-close	I-Close	None
2	laparoscopic	SAA	SAA	I-Close	None
3	laparoscopic	SAA	SAA	Not closed	None
4	laparoscopic	SAA	SAA	I-Close	Biliary enteric anastomotic leak
5	open	SAA	SAA	I-Close	None
6	open	SAA	SAA	Not closed	None
7	laparoscopic	SAA	SAA	Not closed	None
8	laparoscopic	SAA	SAA	I-Close	Biliary enteric anastomotic leak
9	laparoscopic	SAA	SAA	Not closed	Biliary enteric anastomotic leak

SAA, same as above; C-Close, continuously closed with absorbable sutures; I-Close, intermittently closed with absorbable sutures.

### 3.2 Intestinal obstruction surgery details

The perioperative details for the intestinal obstruction surgeries are summarized in Table 2. Among the 9 patients, 8 were female, and 1 was male, with ages ranging from 2 to 14 years, most of whom presented with abdominal pain and vomiting. The interval between the two surgeries ranged from 20 days to 8.84 years, and the duration of conservative treatment ranged from 5 h to 11 days. Surgical indications were as follows: 3 cases showed signs of peritonitis; 4 cases had worsening abdominal pain; and 2 cases had unresolved bowel obstruction after more than 10 days of conservative treatment. Surgical approaches included: 2 patients underwent laparoscopic adhesiolysis (one converted to open surgery); 3 patients underwent reduction of internal hernia, resection of necrotic biliary-jejunal loop, and re-anastomosis of the hepaticojejunostomy; 1 patient received adhesiolysis, reduction, and fixation of the twisted biliary-jejunal loop; 1 patient underwent release and reduction of a Petersen hernia with closure of the defect; and 2 patients had reduction and closure of the transverse mesocolic defect. The causes of obstruction found during surgery included: 2 cases of adhesions or band compression, 6 cases of internal hernias (2 Petersen hernias, 3 mesocolic defect hernias, 1 Brolin hernia), and 1 case of a 720° rotation of the the biliary-jejunal loop. All surgeries involved careful exploration and closure of the Petersen space, Brolin space, and mesocolic defects.

**Table 2:** Perioperative data related to intestinal obstruction surgery.

Case No.	Gender	Age (years)	Symptoms	Case No.	Operative Indication	Surgical Method	Cause of Intestinal Obstruction	Conservative Treatment Time (hours)	Interval Between Two Surgeries (years)
1	Female	7	Abdominal pain, vomiting	1	Aggravated abdominal pain	Intestinal adhesion release and reduction with fixation of the biliary-enteric loop torsion	Torsion of the biliary-enteric loop below the transverse colon by 720°, presenting as a cord-like structure	21	7.69
2	Female	5	Abdominal pain, vomiting	2	Aggravated abdominal pain	Laparoscopic intestinal adhesion release	Compression of the terminal ileum by a band	30	0.12
3	Female	3	Abdominal pain, vomiting	3	Aggravated abdominal pain	Release and reduction of internal hernia and closure of the mesocolic defect of the transverse colon	Small intestine herniates through the mesocolic defect, with the herniated segment located 130 cm from the cecum	40	0.05
4	Female	2	Abdominal pain, vomiting	4	No relief of symptoms after 11 days of conservative treatment	Laparoscopic exploration and adhesiolysis (converted to open surgery)	Band entrapment of the intestinal segment at the root of the mesentery, leading to obstruction	264	0.34
5	Female	14	Abdominal pain	5	Peritonitis in the upper abdomen	Release and reduction of internal hernia,resection of necrotic biliary-jejunal loop, and re-anastomosis of the hepaticojejunostomy	The upper segment of the jejunum, along with the side-to-side intestinal anastomosis, herniated into the Petersen's space, leading to a 720-degree rotation of the biliary-enteric loop and necrosis	5	8.84
6	Female	7	Abdominal pain, bloating, vomiting	6	Peritonitis throughout the abdomen	Same As Above	Herniation of the biliary-enteric loop into the Brolin's space, with complete necrosis of the biliary-enteric loop and the intestinal segment at the end-to-side anastomosis	15	1.58
7	Female	6	Abdominal pain, vomiting	7	Upper abdominal peritonitis	Same As Above	Herniation of the proximal jejunum, including the end-to-side anastomosis of jejunum, through the mesocolic defect of the transverse colon, compressing and causing necrosis of the biliary-enteric loop	18	1.6
8	Female	3	Abdominal pain, vomiting	8	No relief of symptoms after 10 days of conservative treatment	Release and reduction of internal hernia and closure of the Petersen's space	Herniation of the intestinal segment, 120 cm from the cecum, into the Petersen's space	240	0.91
9	Male	2	Abdominal pain, vomiting	9	Aggravated abdominal pain	Release and reduction of internal hernia and closure of the mesocolic defect of the transverse colon	Herniation of the intestinal segment, 100 cm from the cecum, into the mesocolic defect of the transverse colon	69	0.92

### 3.3 Preoperative imaging and laboratory data for intestinal obstruction surgery

**Figure 2:**
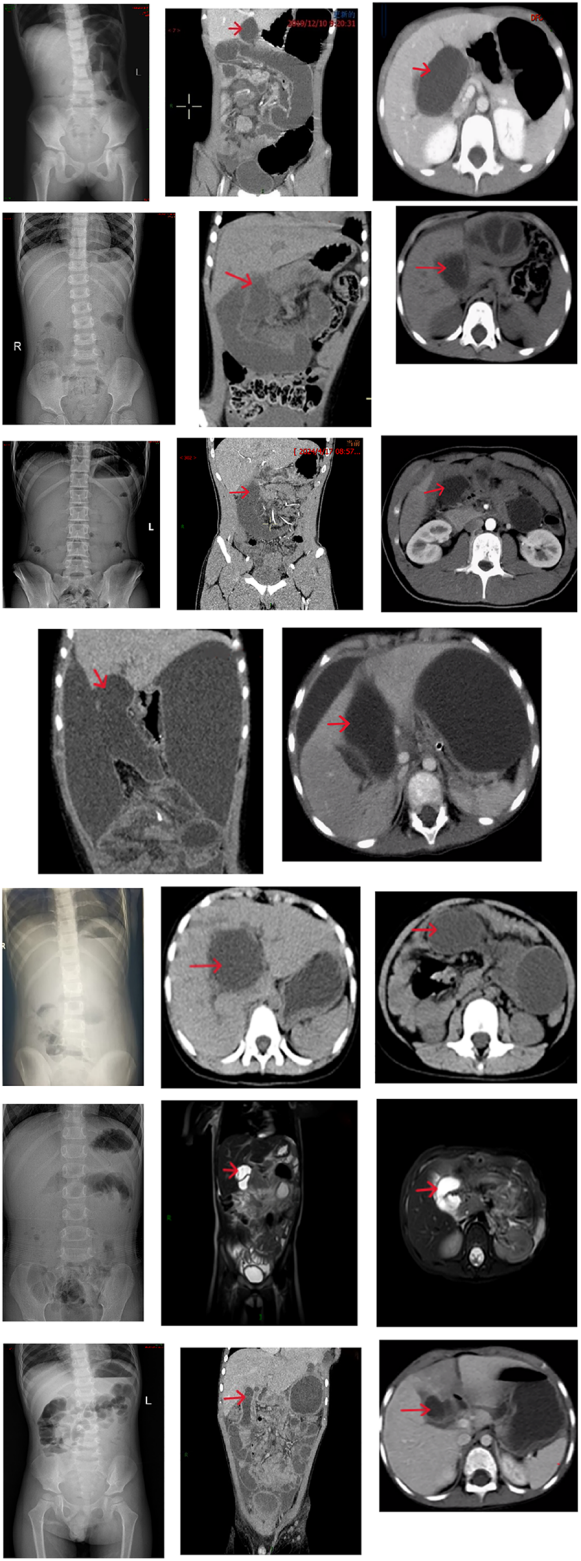
Case 1: The bile duct Y-loop is twisted 720°, appearing as a cord-like structure. The arrow indicates the dilated bile duct loop at the first hepatic hilum. Case 3: Transverse mesocolic defect hernia (small intestine herniates through the mesocolic defect, with the herniated segment located 130 cm from the cecum). The arrow indicates the dilated bile duct loop at the first hepatic hilum. Case 5: Petersen's hernia (proximal jejunum, including the jejunojejunal anastomosis, herniates into the Petersen's space causing a 720° rotation of the bile duct loop and necrosis of the bile duct loop). The arrow indicates the dilated bile duct loop at the first hepatic hilum. Case 6: Brolin hernia (the bile duct loop herniates into the Brolin's space, with complete necrosis of the biliary-jejunal loop and the biliary-jejunal loop jejunojejunal anastomosis). The patient did not undergo an abdominal x-ray preoperatively. The arrow indicates the dilated bile duct loop at the first hepatic hilum. Case 7: Transverse colonic mesenteric defect hernia (the proximal jejunum, including the jejunojejunal anastomosis, herniates into the colonic mesenteric defect, resulting in necrosis of the bile duct loop). This patient underwent intestinal obstruction surgery at a medical alliance hospital and no coronal CT images were available. The arrow indicates the dilated bile duct loop at the first hepatic hilum. Case 8: Petersen's hernia (herniation of the intestinal segment, 120 cm from the cecum, into the Petersen's space). The patient did not undergo abdominal CT examination. The arrow indicates the dilated bile duct loop at the first hepatic hilum. Case 9: Colonic mesenteric defect hernia (herniation of the intestinal segment, 100 cm from the cecum, into the mesocolic defect of the transverse colon). The arrow indicates the dilated bile duct loop at the first hepatic hilum.

Preoperative laboratory and imaging data are summarized in Table 3. Two patients demonstrated conjugated hyperbilirubinemia on serial liver function. Abdominal x-ray in the standing position showed decreased bowel gas or small air-fluid levels in 8 patients. Abdominal CT or MRI revealed signs of bowel obstruction, volvulus, internal hernia, and bowel necrosis. In patients diagnosed intraoperatively with internal hernias or bowel volvulus, preoperative CT or MRI identified fluid accumulation in the biliary-jejunal loop at the hepatic hilum, as shown in Figure 2 (cases 1, 3, 5, 6, 7, 8, and 9).

**Table 3:** Preoperative imaging and liver function in patients with intestinal obstruction.

Case No.	Upright abdominal x-ray	Abdominal and Pelvic CT/MRI Findings	Total Bilirubin (µmol/L)	Direct Bilirubin (µmol/L)	Gamma-Glutamyl Transferase (U/L)	Alkaline Phosphatase (U/L)
1	Left-sided intestinal distention; multiple small air-fluid levels	Intestinal obstruction, possible volvulus and necrosis;dilated bowel in the hepatic hilum	134	78.1	104	185
2	Mild distention of the bowel in the upper abdome	No significant organic disease in abdominal solid organ;No signs of intestinal obstruction	7.4	2	13	148
3	Slight air accumulation in the bowel	Small bowel dilation with fluid accumulation is observed in the upper abdomen, raising the possibility of an internal hernia	7.1	2.8	10	141
4	Several small air-fluid levels in the upper abdomen	Intestinal obstruction with portal venous air. Dilated bowel loops are seen in the hepatic hilum	3.6	1.2	8.5	111
5	No significant air-fluid levels or abnormal gas-filled loops visible in the abdomen	Intestinal obstruction with volvulus, along with intra-abdominal and pelvic fluid accumulation; dilated bowel loops in the hepatic hilum	62.3	19.1	11.6	144
6	None	Slight dilation of the bile ducts at the hepatic hilum, with abnormally dilated bowel loops in the hepatic hilum and massive ascites	16.1	8.6	11.7	60
7	Short fluid level in the abdomen	Massive multilocular lesion in the abdominal cavity; dilated bowel loops in the hepatic hilum	13.6	5	23.9	166
8	No significant air-fluid levels or abnormal gas-filled loops visible in the abdomen	Dilated bowel loops with fluid accumulation at the hepatic hilum	7.4	1.9	10.5	183
9	Slight gas accumulation in the middle abdomen, small fluid level	Small bowel volvulus cannot be excluded, with prominent dilated bowel loops at the hepatic hilum and fluid accumulation	9.6	4.4	10	127

Reference ranges: Total Bilirubin: ≤21 µmol/L, Direct Bilirubin: ≤8 µmol/L,Gamma-Glutamyl Transferase: ≤19 U/L,Alkaline Phosphatase: ≤406 U/L.

### 3.4 Follow-up after surgery for intestinal obstruction

The postoperative course was uneventful in all cases. Through December 1, 2024, comprehensive clinical follow-up demonstrated maintenance of normal hematological indices, hepatic function, hepatobiliary ultrasound findings, and age-appropriate developmental progress.

## 4 Discussion

CBD is a congenital biliary disorder in children. Although surgical treatment generally achieves favorable outcomes, complications such as bilioenteric anastomotic stricture, pancreatitis, reflux cholangitis, and intestinal obstruction are not uncommon. Among these, intestinal obstruction remains underexplored in pediatric populations. In this study, we retrospectively analyzed the treatment and prognosis of 475 pediatric patients who underwent surgical management for CBD at our center over the past 11 years. Notably, 9 patients developed postoperative intestinal obstruction that required surgical intervention. Comprehensive clinical data were collected for these cases, encompassing both the initial definitive surgery and the subsequent intestinal obstruction operation. This analysis highlights the diagnostic and therapeutic characteristics of intestinal obstruction following surgery for CBD in children, addressing gaps in systematic research on this topic.

### 4.1 Causes and incidence of intestinal obstruction

Previous studies have indicated that common causes of intestinal obstruction following definitive surgery for CBD include internal hernia, adhesive intestinal obstruction, and biliary-jejunal loop obstruction (4–6). Following retrocolic Roux-en-Y hepaticojejunostomy, three potential mesenteric defects may arise: Petersen's space, Brolin's space, and the transverse colon mesenteric defect (7). In our cohort, all internal hernias occurred through these three mesenteric defects. Previous studies have reported an incidence of adhesive intestinal obstruction after CBD surgery ranging from 3% to 4% (8, 9), and biliary-jejunal loop obstruction occurs at a rate of 0.8% (5). In our center, the incidence rates for adhesive obstruction, biliary-jejunal loop obstruction, and internal hernias were 0.4%, 0.2%, and 1.5%, respectively. Notably, the incidence of adhesive obstruction was significantly lower than previously reported, likely due to our study's inclusion criteria, which focused only on patients undergoing surgical treatment for adhesive obstruction and excluded those managed conservatively. The relatively high rate of internal hernias (66.7%) in this cohort suggests that, following CBD surgery, internal hernias are the most frequent cause of intestinal obstruction. This emphasizes the necessity of a diagnostic and therapeutic strategy tailored to intestinal obstruction following Roux-en-Y hepaticojejunostomy, rather than relying solely on principles for managing adhesive obstruction (10).

Biliary-jejunal loop obstruction is a specific type of intestinal blockage that occurs after Roux-en-Y hepaticojejunostomy. We identify four major types of biliary-jejunal loop obstruction: (1) Axial rotation of the Roux limb (e.g., Case 1); (2) Herniation of bowel into the Petersen's space or mesocolic defect of the transverse colon, leading to obstruction or subsequent torsion of the biliary-jejunal loop (e.g., Cases 5 and 7), both of which resulted in biliary-jejunal loop necrosis; (3) Herniation of the biliary-jejunal loop into the Brolin's space (e.g., Case 6); (4) Herniation of the biliary-jejunal loop through the mesocolic defect of the transverse colon, causing obstruction. Although such cases have been reported in the literature (10), they were not observed in our cohort. These conditions all fall under the category of closed-loop intestinal obstruction, often resulting in more severe complications, such as biliary-jejunal loop necrosis, necessitating prompt surgical intervention.

In light of the findings, we propose the “internal hernia triangle” as a high-risk area for internal hernia after Roux-en-Y hepaticojejunostomy. This triangle is delineated by the duodenojejunal junction anchored by the ligament of Treitz, the transverse mesocolic defect, and the site of jejunal anastomosis. It includes all three mesenteric defects: the Brolin's space on the lower left, the Petersen's space on the right, and the mesocolic defect on the upper right (Figure 1). The triangular area is a common site for internal hernias. In the present study, all six internal hernias occurred in this triangle, suggesting that when intestinal obstruction is suspected after CBD surgery, special attention should be paid to this region on CT imaging. If abnormalities such as bowel distension, mesenteric irregularity, or the “whirl sign” are observed within this region, internal hernia should be highly suspected (11). Intraoperatively, the triangle should be prioritized for exploration to reduce operative time and minimize tissue trauma.

### 4.2 Diagnosis and management principles

All patients in the study presented with abdominal pain and vomiting, which required differentiation from conditions such as biliary anastomotic stricture, reflux cholangitis, acute pancreatitis, and acute gastroenteritis. After excluding these diagnoses, the possibility of intestinal obstruction secondary to CBD surgery should be considered. Abdominal x-ray is a common tool for the initial diagnosis of intestinal obstruction. In this research, 8 patients underwent preoperative abdominal x-ray, which showed decreased or uneven gas distribution and small bowel fluid levels, but without clear signs of obstruction. Previous studies have also demonstrated the limited value of abdominal x-rays in diagnosing internal hernias (12), which is consistent with our findings. Thus, abdominal x-ray can serve as an initial screening tool, but its results should be interpreted cautiously, especially in patients with Roux-en-Y biliary reconstruction. Abdominal CT is more valuable in evaluating abdominal pain and vomiting following Roux-en-Y reconstruction, especially when complications such as internal hernia need to be excluded (13, 14). Imaging plays a crucial role in identifying the cause of intestinal obstruction and determining the appropriate treatment. Adhesive intestinal obstruction may be managed conservatively with a reasonable success rate (15). However, when the obstruction involves the biliary-jejunal loop or internal hernia, surgical intervention is required. In this study, distention and intraluminal fluid retention of the biliary-jejunal loop adjacent to the porta hepatis were frequently observed on preoperative CT or MRI in patients with internal hernia or Roux limb obstruction. Anatomically, hernia contents entering mesocolic defects of the transverse colon, Petersen's space, or Brolin's space can compress the biliary-jejunal loop, causing dilation and fluid accumulation in the Roux limb at the hepatic hilum. Volvulus or obstruction of the biliary loop may have similar effects (16). Theoretically, any obstruction below the bowel-to-bowel anastomosis can lead to distension of the bowel proximal to the obstruction, including the biliary-jejunal loop. In this research, two cases of adhesive obstruction occurred distal to the anastomosis, but abdominal CT did not show distended and fluid-filled Roux limb near the porta hepatis, possibly due to gastrointestinal decompression following the placement of a gastric tube, which does not relieve distension or fluid accumulation of the biliary-jejunal loop in conditions of internal hernia or Roux limb obstruction. The detection of biliary-enteric loop dilatation and intraluminal fluid retention on CT imaging at the porta hepatis is strongly suggestive of Roux limb obstruction or internal herniation. Given the pathognomonic nature of these findings, prompt surgical intervention is recommended to mitigate risks of progressive luminal compromise or ischemic sequelae. Although no standardized quantitative criteria exist for objectively assessing these findings, we propose using the descending duodenum as an internal reference. If the biliary-enteric loop at the hepatic hilum is significantly wider than the descending duodenum, it may indicate distention. In our cohort, surgical indications were exacerbated abdominal pain (4 cases), signs of peritonitis (3 cases), and failure to resolve with conservative treatment (2 cases). In the three cases with peritonitis, biliary-jejunal loop necrosis was confirmed during surgery. This suggests that surgery should not be delayed until peritoneal signs emerge, as these may indicate severe complications such as bowel necrosis. When abdominal pain worsens, CT reveals dilation and fluid accumulation in the biliary-jejunal loop at the porta hepatis, along with abnormal imaging in the internal hernia triangle, surgical exploration should be considered. As stated in the literature, timely surgical intervention is often more effective than delayed observation (12).

### 4.3 Prevention of intestinal obstruction

During the initial surgery, efforts should focus on ensuring high-quality bilioenteric anastomosis, preventing bile leakage, and minimizing peritoneal contamination from bile and intestinal contents, all of which help reduce the risk of adhesive bowel obstruction. A meta-analysis has demonstrated that Seprafilm significantly reduces the incidence of postoperative adhesive intestinal obstruction following abdominal surgery, thereby supporting its routine application as a prophylactic adjunct in surgical practice (17). Randomized controlled studies have suggested that routinely closing mesenteric defects after Roux-en-Y reconstruction can reduce the incidence of internal hernias (18). Notably, one case of Brolin hernia occurred in the cohort despite definitive intraoperative closure of the Brolin space during the initial surgical procedure. This observation underscores that even rigorous adherence to mesenteric defect closure protocols may not entirely eliminate the risk of internal herniation (19). Generally, during defect closure, minimal mesenteric tissue is sutured to avoid vascular injury, yet this approach inherently reduces tensile strength of the repaired tissue, predisposing to dehiscence. Persistent intestinal motility may disrupt nascent adhesions, leading to incomplete defect closure and recurrent dehiscence. These observations collectively emphasize the necessity for vigilant postoperative surveillance for internal hernias, even after technically adequate primary closure. One patient in our series developed spontaneous biliary-jejunal loop torsion within the infracolic compartment. Current literature describes similar torsion events in supracolic positions (4), with excessive loop length identified as a key risk factor. To mitigate this, individualized loop construction—tailoring biliary-jejunal loop length according to hepatoportal-umbilical distance—has demonstrated efficacy in preventing torsion (20, 21), representing a clinically adoptable strategy.

## 5 Conclusion

Postoperative intestinal obstruction following excision of the dilated bile duct and Roux-en-Y hepaticojejunostomy in pediatric patients is primarily caused by internal hernias, adhesions, or biliary-jejunal loop torsion, with internal hernias being the most common, particularly located in the “hernia triangle”. Abdominal x-ray has limited diagnostic value, while abdominal CT or MRI showing fluid accumulation and distension in the biliary-jejunal loop near the porta hepatis strongly suggests internal hernia or biliary-jejunal loop torsion. Given the higher likelihood of internal hernia and its rapid progression to Roux limb necrosis, early surgical intervention should be considered.

## 6 Limitations of the study

This study has several limitations. Firstly, the descriptive design—characterized by a small sample size and lack of comparative groups—limits the generalizability of findings and reduces the level of evidence. Furthermore, we were unable to provide intraoperative imaging to substantiate our findings, All descriptions lacked intraoperative photographic support, which weakens the strength of our conclusions.

## Data Availability

The datasets presented in this study can be found in online repositories. The names of the repository/repositories and accession number(s) can be found in the article/Supplementary Material.

## References

[1] Soares KC Kim Y Spolverato G Maithel S Bauer TW Marques H Presentation and clinical outcomes of choledochal cysts in children and adults. JAMA Surg. (2015) 150:577. 10.1001/jamasurg.2015.0226 25923827

[2] Ishibashi H Shimada M Kamisawa T Fujii H Hamada Y Kubota M Japanese clinical practice guidelines for congenital biliary dilatation. J Hepatobiliary Pancreat Sci. (2017) 24:1–16. 10.1002/jhbp.415 28111910

[3] Kim Y Crookes PF. Complications of bariatric surgery. In: Huang C-K, editor. Essentials and Controversies in Bariatric Surgery. Rijeka: IntechOpen (2014). p. Ch. 3.

[4] Tang S-T Yang Y Wang Y Mao YZ Li SW Tong QS Laparoscopic choledochal cyst excision, hepaticojejunostomy, and extracorporeal Roux-en-Y anastomosis: a technical skill and intermediate-term report in 62 cases. Surg Endosc. (2011) 25:416–22. 10.1007/s00464-010-1183-y 20602140

[5] Qiao G Li L Li S Tang S Wang B Xi H Laparoscopic cyst excision and Roux-Y hepaticojejunostomy for children with choledochal cysts in China: a multicenter study. Surg Endosc. (2015) 29:140–4. 10.1007/s00464-014-3667-7 25125091

[6] Lee KH Tam YH Yeung CK Chan KW Sihoe JD Cheung ST Laparoscopic excision of choledochal cysts in children: an intermediate-term report. Pediatr Surg Int. (2009) 25:355–60. 10.1007/s00383-009-2343-9 19255762

[7] Fry BT Finks JF. Abdominal pain after Roux-en-Y gastric bypass. JAMA Surg. (2023) 158:1096. 10.1001/jamasurg.2023.3211 37531117

[8] Matsumoto M Urushihara N Fukumoto K Yamoto M Miyake H Nakajima H. Laparoscopic management for prenatally diagnosed choledochal cysts. Surg Today. (2016) 46:1410–4. 10.1007/s00595-016-1319-3 26935547

[9] Urushihara N Fukumoto K Fukuzawa H Mitsunaga M Watanabe K Aoba T Long-term outcomes after excision of choledochal cysts in a single institution: operative procedures and late complications. J Pediatr Surg. (2012) 47:2169–74. 10.1016/j.jpedsurg.2012.09.001 23217870

[10] O’Rourke RW. Management strategies for internal hernia after gastric bypass. J Gastrointest Surg. (2011) 15:1049–54. 10.1007/s11605-010-1401-x 21547708

[11] Ederveen JC Nienhuijs SW Jol S Robben SGF Nederend J. Structured CT reporting improves accuracy in diagnosing internal herniation after laparoscopic Roux-en-Y gastric bypass. Eur Radiol. (2020) 30:3448–54. 10.1007/s00330-020-06688-x 32078011 PMC7248015

[12] Khanna A Newman B Reyes J Fung JJ Todo S Starzl TE. Internal hernia and volvulus of the small bowel following liver transplantation. Transpl Int. (1997) 10:133–6. 10.1111/j.1432-2277.1997.tb00555.x 9089999 PMC3005197

[13] Al-Mansour MR Mundy R Canoy JM Dulaimy K Kuhn JN Romanelli J. Internal hernia after laparoscopic antecolic Roux-en-Y gastric bypass. Obes Surg. (2015) 25:2106–11. 10.1007/s11695-015-1672-0 26037306

[14] Greenstein AJ O’Rourke RW. Abdominal pain after gastric bypass: suspects and solutions. Am J Surg. (2011) 201:819–27. 10.1016/j.amjsurg.2010.05.007 21333269 PMC3123682

[15] Singhavejsakul J Ukarapol N. Choledochal cysts in children: epidemiology and outcomes. World J Surg. (2008) 32:1385–8. 10.1007/s00268-008-9582-0 18408962

[16] Kawkabani Marchini A Denys A Paroz A Romy S Suter M Desmartines N The four different types of internal hernia occurring after laparascopic Roux-en-Y gastric bypass performed for morbid obesity: are there any multidetector computed tomography (MDCT) features permitting their distinction? Obes Surg. (2011) 21:506–16. 10.1007/s11695-011-0364-7 21318275

[17] Hajibandeh S Hajibandeh S Saeed S Bird J Kannappa L Ratnayake I. Effect of hyaluronate-based bioresorbable membrane (Seprafilm) on outcomes of abdominal surgery: a meta-analysis and trial sequential analysis of randomised controlled trials. Updates Surg. (2022) 74:865–81. 10.1007/s13304-021-01117-0 34148173

[18] Stenberg E Ottosson J Magnuson A Szabo E Wallén S Näslund E Long-term safety and efficacy of closure of mesenteric defects in laparoscopic gastric bypass surgery. JAMA Surg. (2023) 158:709. 10.1001/jamasurg.2023.1042 37163240 PMC10173104

[19] Altieri MS Carter J Aminian A Docimo S Jr Hinojosa MW Cheguevara A American Society for metabolic and bariatric surgery literature review on prevention, diagnosis, and management of internal hernias after Roux-en-Y gastric bypass. Surg Obes Relat Dis. (2023) 19:763–71. 10.1016/j.soard.2023.03.019 37268518

[20] Diao M Li L Zhang J-Z Cheng W. A shorter loop in Roux-Y hepatojejunostomy reconstruction for choledochal cysts is equally effective: preliminary results of a prospective randomized study. J Pediatr Surg. (2010) 45:845–7. 10.1016/j.jpedsurg.2009.12.022 20385300

[21] Baba A Yamazoe S Dogru M Okuyama Y Mogami T Kobashi Y Petersen hernia after open gastrectomy with Roux-en-Y reconstruction: a report of two cases and literature review. SpringerPlus. (2015) 4:753. 10.1186/s40064-015-1556-8 26693111 PMC4666877

